# Identification and Regulatory Network Analysis of Genes Related to Reproductive Performance in the Hypothalamus and Pituitary of Angus Cattle

**DOI:** 10.3390/genes13060965

**Published:** 2022-05-27

**Authors:** Yuwen Huang, Chenfeng Yuan, Yun Zhao, Chunjin Li, Maosheng Cao, Haobang Li, Zijiao Zhao, Ao Sun, Wangdui Basang, Yanbin Zhu, Lu Chen, Fang He, Cheng Huan, Boqi Zhang, Tariq Iqbal, Yamen Wei, Wenjing Fan, Kangle Yi, Xu Zhou

**Affiliations:** 1 Jilin Provincial Key Laboratory of Animal Embryo Engineering, College of Animal Science and Veterinary Medicine, Jilin University, 5333 Xi’an Avenue, Changchun 130062, China; huangyw18@mails.jlu.edu.cn (Y.H.); yuancf20@mails.jlu.edu.cn (C.Y.); zhao_yun@jlu.edu.cn (Y.Z.); llcjj158@163.com (C.L.); caoms18@mails.jlu.edu.cn (M.C.); zhaozj18@mails.jlu.edu.cn (Z.Z.); luchen@jlu.edu.cn (L.C.); zhangbq19@mails.jlu.edu.cn (B.Z.); iqbaltariq9917@mails.jlu.edu.cn (T.I.); weiym19@mails.jlu.edu.cn (Y.W.); fanwj19@mails.jlu.edu.cn (W.F.); 2 Hunan Institute of Animal and Veterinary Science, 8 Changliang Road, Changsha 410131, China; lhb.m2002@163.com (H.L.); kkkkanty@163.com (A.S.); hf3893@126.com (F.H.); rachel_613@163.com (C.H.); 3 Laboratory of Hulless Barley and Yak Germplasm Resources and Genetic Improvement, Lhasa 850002, China; bw0891@163.com (W.B.); zhuyanbin163@163.com (Y.Z.)

**Keywords:** angus cattle, high-throughput sequencing, pituitary, hypothalamus, reproductive performance

## Abstract

In this study, we explored the gene expression patterns of the pituitary gland and hypothalamus of Angus cows at different growth and developmental stages by deep sequencing and we identified genes that affect bovine reproductive performance to provide new ideas for improving bovine fertility in production practice. We selected three 6-month-old (weaning period), three 18-month-old (first mating period), and three 30-month-old (early postpartum) Angus cattle. The physiological status of the cows in each group was the same, and their body conformations were similar. After quality control of the sequencing, the transcriptome analyses of 18 samples yielded 129.18 GB of clean data. We detected 13,280 and 13,318 expressed genes in the pituitary gland and hypothalamus, respectively, and screened 35 and 50 differentially expressed genes (DEGs) for each, respectively. The differentially expressed genes in both tissues were mainly engaged in metabolism, lipid synthesis, and immune-related pathways in the 18-month-old cows as compared with the 6-month-old cows. The 30-month-old cows presented more regulated reproductive behavior, and pituitary CAMK4 was the main factor regulating the reproductive behavior during this period via the pathways for calcium signaling, longevity, oxytocin, and aldosterone synthesis and secretion. A variant calling analysis also was performed. The SNP inversions and conversions in each sample were counted according to the different base substitution methods. In all samples, most base substitutions were represented by substitutions between bases A and G, and the probability of base conversion exceeded 70%, far exceeding the transversion. Heterozygous SNP sites exceeded 37.68%.

## 1. Introduction

As the demand for beef increases, beef cattle are distributed worldwide. Angus cattle are a superior breed of beef cattle owing to their excellent meat performance and they are considered to be a typical specialized breed of beef cattle worldwide. In addition to their delicious meat, Angus cattle exhibit good lactation ability. Because of the significant advantages of Angus cattle, research on this breed continues. Angus cattle growth and development has been well studied [1]. Angus cattle are normally weaned at 6 months old and gradually reach sexual maturity at 12–14 months old. Primary mating can occur at 18 months of age, and the primiparity usually occurs at 30 months of age. Factors affecting the pregnancy rates and lactation of dairy cows and the relationship between the two have been discussed, and various methods for improving reproductive performance have been proposed [2,3]. However, for Angus cattle, the genes which cause the differences in reproductive performance at three different growth and development stages are unknown.

The pituitary-hypothalamus-gonadal (HPG) axis stems from interaction between nerve and hormone signals from three main sources. Through the release of gonadotropin-releasing hormone (GnRH) and gonadotropin-inhibitory hormone (GnIH), the hypothalamus acts on the pituitary gland to regulate gonadotropins and secrete the pituitary hormone, luteinizing hormone (LH). prolactin (PRL) and follicle-stimulating hormone (FSH) are involved in oocyte maturation, corpus luteum formation, and ovulation. These hormones act on the gonads and regulate steroid hormone production. Interactions among these three signals ensures that the HPG axis regulates reproductive activity. Ovulation, fertilization, and lactation are the keys to successful reproduction, and sexual maturity of the gonad organs helps ensure these behaviors. However, in dairy cattle, substantial genetic antagonism may exist between high yield and fertility in dairy cattle, in other words, high milk production is often accompanied by low fertility [4] Although prostaglandin and GnRH are combined early to induce ovulation to promote pregnancy and alleviate this dilemma, the mechanism behind this remains unclear. Comparing the different sexual development stages of dairy cows may provide clues to understanding this phenomenon. Early research on dairy cows showed that body quality scores during calving and early lactation are related to milk quantity. Few studies on beef cattle have been published in this area, and insufficient evidence exists to show a connection; however, studies have found that milk from beef cattle influenced these mechanisms differently from that of other cows [5].

With the development of high-throughput sequencing technology, transcriptomics research has become increasingly widely used to systematically study genes involved in biological processes and developmental stages, as well as responses to biological changes [6]. The establishment and continuous improvement of annotation information of the bovine genome [7] has progressed the use of transcriptomics to study cattle-related issues. Next-generation RNA sequencing technology applied to cattle will affect their short-term dietary control, immune responses, neuroprotection, energy homeostasis, and eventually, their estrus cycles. In addition to exploring the external factors that affect bovine estrus, sequencing technology is being used to determine the differences in HPG axis levels before and after estrus. Conjoint analysis of five reproductive tissues (i.e., the hypothalamus, pituitary, ovary, uterus, and endometrium) and some growth- and metabolism-related tissues with genome-wide association studies, transcriptomes, and bovine transcription factors have enabled the study of co-expression networks around puberty [8].

Beef cattle breeding is an important part of the animal husbandry industry, which has a significant impact on the development of the agriculture and animal husbandry economies and the improvement of national diet structure. Beef is favored by consumers because of its high lean meat rate and low fat. Angus cattle are one of the three high quality beef cattle breeds, with good meat production performance, high meat rate of quality characteristics, and is a relatively common breed of beef cattle breeding. We collected the hypothalamus and pituitary tissues from three stages of sexual development and explored the regulatory mechanisms guiding sexual maturation in angus cattle gonadal development via high-throughput RNA sequencing technology to further resolve existing problems in production practice.

## 2. Materials and Methods

### 2.1. Animal Tissue Collection

Nine Angus cattle at the ages of 6, 18, and 30 months from the Changsha Institute of Animal Science were selected and divided into 3 groups according to age, with 3 cows per group. Six-month-old cows are freshly weaned calves, 18-month-old cows are sexually mature cows that meet the requirements for priming, and 30-month-old cows have completed producing their first progeny. These cattle were maintained in similar housing and feeding conditions, the selected cattle were healthy and disease free, and their body weights and physiological states were similar in each group. External observation and rectal ovarian and uterine evaluation before slaughter confirmed that all cattle were not in heat. After evaluation, the cattle were stunned with an electric shock, and then euthanized by carotid bloodletting. After the cattle was completely dead, the upper hypothalamus and pituitary glands of the cattle were collected, frozen in liquid nitrogen until RNA isolation, and numbered according to age and tissue type. Supplementary Table S1 shows the numbering and grouping information. All animal protocols were carried out according to the guidance of the Animal Care Committee at Jilin University. All ethical assessments were carried out in accordance with the requirements of China’s Laboratory Animal Management Regulations.

### 2.2. RNA Extraction and cDNA Library Preparation

The collected tissue samples were processed by grinding in liquid nitrogen, and the total RNA was extracted using the TRIzol protocol (Invitrogen, Carlsbad, CA, USA). The RNA concentration was measured using an ultramicro spectrophotometer (Tuohe, Shanghai, China) and the RNA quality was evaluated using the RNA integrity number (RIN) value from an Experion automated electrophoresis system (BioRad, Hercules, CA, USA). To make sure there was enough mRNA to build the cDNA library, the total RNA of each sample needed to be ≥4.0 μg, and the RIN values needed to be ≥8.0. The PolyATract mRNA separation system (Promega, Madison, WI, USA) was used to further isolate the poly-a-containing mRNA from each sample of total RNA. The mRNA was fragmented, then the reverse transcriptase and random primers were used to convert RNA fragments into first-strand cDNA. DNA polymerase I and RNase H were used to synthesize the second-strand cDNA, The cDNA sticky ends were repaired into flat ends using T4 DNA polymerase and Klenow DNA polymerase, and the cDNA 3′ ends were added base A, then, the adapters were ligated. Next, the product was purified using an AMPure XP system, and PCR amplification yielded the final cDNA library. An Aligent Technologies 2100 bioanalyzer (Agilent, Palo Alto, CA, USA) was used to assess the quality of the cDNA library.

### 2.3. Data Analysis

#### 2.3.1. Quality Control

Raw data of fastq format were firstly processed through in-house perl scripts. In this step, clean reads were obtained by removing reads containing adapter, ploy-N, and low-quality reads from the raw data. At the same time, Q20, Q30, GC content, and sequence duplication level of the clean data were calculated. The quality score was integer mapping of the probability of base identification errors, and the Phred base quality value formula was commonly used: $Q = −10 × log {10}P$, P was the probability of base identification error, and the corresponding relationship between base mass value and the probability of identification error was: Q20 = 1/100, Q30 = 1/1000. All the downstream analyses were based on clean data with high quality (Q30 > 90%, Supplementary Table S2). Then, these clean reads were mapped to the reference genome sequence (Bos taurus reference genome, version UMD 3.1.1); only reads with a perfect match or one mismatch were further analyzed and annotated. The Hisat2 tools software was used to map with reference genome [9].

#### 2.3.2. Gene Functional Annotation

Gene function was annotated based on the following databases: Nr (NCBI non-redundant protein sequences) [10], Nt (NCBI non-redundant nucleotide sequences) [11], Pfam (protein family) [12], KOG/COG (clusters of orthologous groups of proteins) [13,14], Swiss-Prot (a manually annotated and reviewed protein sequence database) [15], KO (KEGG Ortholog database) [16], and GO (gene ontology) [17].

#### 2.3.3. SNP (Single Nucleotide Polymorphisms) Calling

Picard tools v1.41 and Samtools v0.1.18 software were used to sort, remove duplicated reads, and merge the bam alignment results of each sample. GATK2 or Samtools software were used to perform SNP calling. Raw vcffiles were filtered with GATK standard filtermethod and other parameters (cluster window size 10, MQ0 >= 4 and (MQ0/(1.0*DP)) > 0.1, QUAL < 10, QUAL < 30.0, or QD < 5.0 or HRun > 5), and only SNPs with distance >5 were retained [18].

#### 2.3.4. Variable Splicing Event Prediction

As described above, cleaned reads from each sample were mapped to the reference genome with HISAT2, StringTie was used for transcript assembly, the variable splicing types and corresponding expression levels of each sample were obtained using ASprofile software (Supplementary Figure S1) [19]. Variable shear was mainly based on the alternative 5′ first exon (TSS) and alternative 3′ last exon (TTS), and a few alternative exons ends or skipped exons were present. Such changes may cause premature termination of the protein coding, thereby acting as a molecular switch (Supplementary Table S3).

#### 2.3.5. Differential Expression Analysis

For the samples with biological replicates, a differential expression analysis of two conditions/groups was performed by DESeq2 [20]. DESeq2 provided statistical routines for determining differential expression in digital gene expression data using a model based on the negative binomial distribution. The resulting *p*-values were adjusted using the Benjamini and Hochberg’s approach for controlling the false discovery rate. Genes with an adjusted *p*-value <0.05 found by DESeq2 were assigned as differentially expressed.

#### 2.3.6. Quantification of Gene Expression Levels

Quantifications of gene expression levels were shown as fragments per kilobase of transcript per million fragments mapped. The formula is shown as follow:

$$FPKM = {cDNA Fragments\over {Mapped Fragments (Millions) * Transcript Length (kb)}} $$

#### 2.3.7. GO Enrichment Analysis

The gene ontology (GO) enrichment analysis of the differentially expressed genes (DEGs) was implemented using the GOseq R packages based on Wallenius noncentral hypergeometric distribution [17], which could adjust for gene length bias in the DEGs. Gene numbers were calculated for every term, significantly enriched GO terms in the DEGs as compared with the genome background were defined using a hypergeometric test. The calculating formula of *p*-value is:

$$P=1-\overset{M}{\underset{i=0}{\sum }}\frac{\left(\begin{array}{c} M\\ i \end{array}\right)\left(\begin{array}{c} N-M\\ n-i \end{array}\right)}{\left(\begin{array}{c} N\\ n \end{array}\right)}$$

where *N* is the number of all genes with GO annotation, n is the number of DEGs in *N*, *M* is the number of all genes that are annotated to the certain GO terms, and m is the number of DEGs in *M*. *N* stands for total background gene (TB gene number), n stands for total significant gene (TS gene number), *M* stands for background gene (B gene number), and m stands for significant gene (S gene number). GO terms meeting this condition with *p* < 0.05 were defined as significantly enriched GO terms in the DEGs. This analysis was able to recognize the main biological functions that the DEGs exercise.

#### 2.3.8. KEGG Pathway Enrichment Analysis

The KOBAS software was used to test the statistical enrichment of differentially expression genes in KEGG pathways [16,21].

#### 2.3.9. PPI (Protein–Protein Interaction)

The sequences of the DEGs were blast (blastx) to the Bos taurus reference genome (version UMD 3.1.1). The PPI information of the DEGs was obtained from the STRING database (http://stringdb.org/ (accessed on 14 March 2021)). Then, the PPI of these DEGs were visualized in Cytoscape [21,22].

### 2.4. Real-Time RT-PCR and Statistical Analysis

The RNA extraction method as described above, and a PrimeScript RT Reagent Kit with gDNA Eraser (Takara, Tokyo, Japan) was used for reverse transcription. RT-qPCR was implemented according to the SYBR Remix Ex TaqII kit. The reaction was performed on a real-time fluorescence quantitative PCR instrument (Mx3005P, Agilent, Santa Clara, CA, USA). The RT-qPCR conditions were as follows: 95 °C for 5 min, (95 °C for 15 s, 58 °C for 20 s, and 72 °C for 20 s) × 40 cycles, 72 °C for 5 min, finally get the melting curve, 95 °C for 15 s, 60 °C for 60 s, and 95 °C for 15 s [23]. Total 20 genes were validated, and the genes are listed in Table 1. The results were analyzed by the 2^−ΔΔCt^ method. The primer sequences of these genes are shown in the Supplementary Table S4; PPP1R11 was the internal reference control gene. All primers were synthesized by the Comate Bioscience Co. Ltd. (Changchun, China). The SPSS version 22.0 (SPSS Inc., Chicago, IL, USA) was used for statistical analysis. The unpaired t-test was used to evaluate the significance of the differences. Statistical significance was indicated at * *p* < 0.05, ** *p* < 0.01, and *** *p* < 0.01. All data were expressed as mean ± SEM.

## 3. Results

### 3.1. Overview of Reads

In the transcriptome analysis of 18 samples, an average of 6.30 GB clean reads were obtained, the GC content was >47.61%, and the ratio of the base percentage of Q30 in each sample was >94.19%. The transcriptome data and reference genome sequence comparison showed that the alignment efficiency of the reads and reference genomes of each sample ranged from 95.06% to 96.53% (Supplementary Table S2), indicating that the selected reference genome assembly met the information analysis needs. The mapped reads in the different regions of the specified reference genome (i.e., exons, introns, and intergenic regions) were counted, and reads from mature mRNAs were compared with exon regions. In this study, 65% of the reads aligned to the exon region, 18.4% of the reads were aligned to the intron region, and because of the imperfect genome annotation, 16.6% of the reads aligned to the intergenic region (Supplementary Figure S2).

### 3.2. SNP/Indel (Insertion and Deletion) Analysis

The GATK software was used to identify base mismatches, insertions, and deletions between the sequenced samples and the reference genome, and then to analyze whether these SNPs affected gene expression levels or protein product types. The SnpEff software was used to annotate and predict the variation effects. Supplementary Tables S2 and S3 list the SNP/Indel site information.

The SNP inversions and conversions in each sample were counted according to the different base substitution methods. In all samples, most base substitutions were represented by substitutions between bases A and G, and the probability of base conversion exceeded 70%, far exceeding the transversion. Heterozygous SNP sites exceeded 37.68% (Supplementary Table S5).

Most SNP densities were concentrated at 0–2 base substitutions per 1000 bp sequence. As the SNP density increases, the number of genes decreases (Supplementary Figure S3a). The SNP/Indel annotation analysis revealed synonymous mutations in each sample, which may be related to altered protein functions (Supplementary Figure S3b,c).

### 3.3. Screening of Gene Expression in the Pituitary Gland and Hypothalamus

Expression level FPKM values ranged from six orders of magnitude 10^−2^ to 10^4^, and the logarithm was mainly concentrated below 1 (Supplementary Figure S4a). To eliminate the effect of heterogeneity in co-expression, we used box plots to more intuitively show the FPKM of each sample (Supplementary Figure S4b). Repeated correlation evaluations were performed on all samples to exclude the biological variability on the results. Pearson’s correlation coefficient (r) was used as the evaluation index of biological repetitive correlations. An r2 closer to 1 means a stronger correlation, as shown in Supplementary Figure S2c; a gradient from purple to blue represents a correlation coefficient from zero to one. The follow-up analysis was performed without abnormal samples (Supplementary Figure S4c). As listed in Supplementary Table S6, DEseq2 was performed on the hypothalamus and pituitary samples from different growth stages to obtain the differentially expressed gene sets between the nine groups of biological conditions.

### 3.4. New Gene Annotation Information in the Pituitary Gland and Hypothalamus

Based on the selected reference genome sequence, the StringTie software was used to splice and quantify mapped reads, which were compared with the original genome annotation information to find the original unannotated transcription regions, discover new transcripts and new genes of the species, and filter out peptide-coding chains that were too short (<50 amino acid residues) or contained only a single exon sequence. A total of 5999 new genes were discovered. A total of 4072 new genes were annotated; 4053 new genes received annotations in NR, 2933 new genes received annotations in GO, 2585 new genes received annotations in eggnog, 2012 new genes received annotations in KEGG (Supplementary Table S7).

### 3.5. Known and Novel Transcript Expression Patterns in the Bovine Pituitary Gland and Hypothalamus

**Figure 1:**
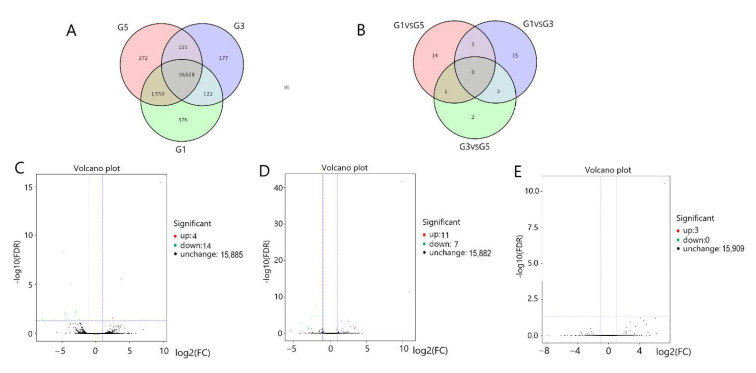
Gene expression patterns in the pituitary glands: (**A**) Gene set at different developmental stages in the pituitary tissue; (**B**) DEGs expressed in the pituitary gland. DEGs expressed in a volcano plot in: (**C**) G3 vs. G1; (**D**) G5 vs. G1; (**E**) G5 vs. G3. G1, G3, G5 = 6-month-old, 18-month-old, 30-month-old Angus cattle pituitary, respectively.

**Figure 2:**
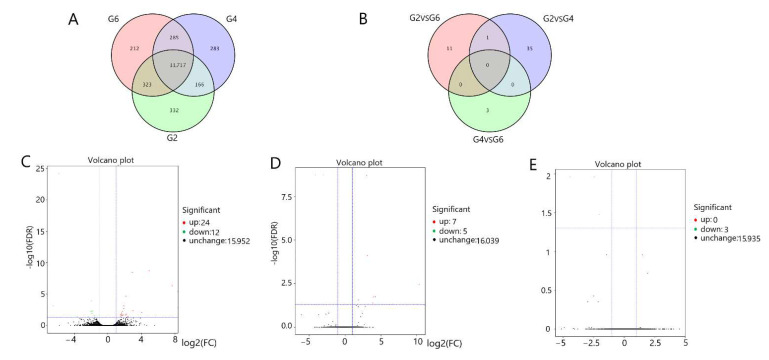
Gene expression patterns in the hypothalamus; (**A**) Gene set at different developmental stages in hypothalamus tissue; (**B**) DEGs expressed in the hypothalamus. DEGs expressed in a volcano plot in: (**C**) G4 vs. G2; (**D**) G6 vs. G2; (**E**) G6 vs. G4. G2, G4, G6 = 6-month-old, 18-month-old, 30-month-old Angus cattle hypothalamus, respectively.

We screened for differentially expressed genes (DEGs) (6-month-old pituitary (G1) vs. 18-month-old pituitary (G3) vs. 30-month-old pituitary (G5); 6-month-old hypothalamus (G2) vs. 18-month-old hypothalamus (G4) vs. 30-month-old hypothalamus (G6)) in the three groups of pituitary and hypothalamus samples. In the pituitary tissues, we detected 13,280 genes and obtained 35 differentially expression genes by pairwise comparison between three parallel groups (Figure 1A,B). As compared with G1, there were 18 DEGs in G3 (4 DEGs in the G3 were upregulated and 14 DEGs were downregulated (Figure 1C)). As compared with G1, there also were 18 DEGs in G5 (11 DEGs in G5 group were upregulated and 7 DEGs were downregulated (Figure 1D)); however, as compared with G3, only 3 DEGs upregulated DEGs in G5 (Figure 1E). In the hypothalamus tissues, we detected 13,318 genes and obtained 50 DEGs by pairwise comparison between three parallel groups (Figure 2A,B). As compared with G2, there also were 36 DEGs in G4 (24 DEGs in G4 group were upregulated and 12 DEGs were downregulated (Figure 2C)); as compared with G2, there were 12 DEGs in G6 (7 DEGs in G6 group were upregulated and 5 DEGs were downregulated (Figure 2D)); as compared with G4, only 3 DEGs downregulated differentially expression genes in G6 (Figure 2E).

### 3.6. Functional Identification of Differentially Expressed Genes in the Pituitary Gland and Hypothalamus

**Figure 3:**
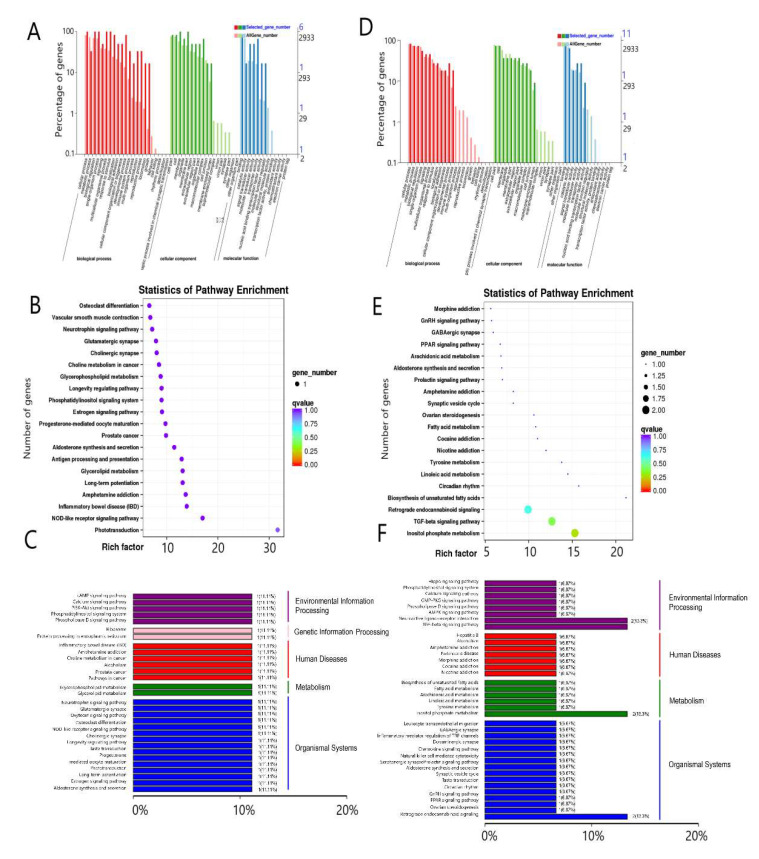
DEG annotation and enrichment in the pituitary and hypothalamus. DEG GO annotation classification chart of the pituitary gland (**A**) and hypothalamus (**D**). DEG scatter plot of KEGG pathway enrichment of the pituitary gland (**B**) and hypothalamus (**E**). DEG KEGG pathway classification of the pituitary gland (**C**) and hypothalamus (**F**).

We performed GO and a KEGG enrichment analysis in the pituitary and hypothalamus tissues. In the three groups of pituitary tissues, six differentially expressed genes significantly enriched biological processes, cellular components, and molecular function. The differentially expressed genes were mainly concentrated in cellular processes (GO:0009987), biological regulation (GO:0065007), membrane portion (GO:0044425), macromolecular complexes (GO:0032991), binding (GO:0005488), and transporter activity (GO:0005215) (Figure 3A). Next, we investigated the KEGG pathways in which all differentially expressed genes were significantly enriched in the pituitary tissues. Thirty enriched terms were involved in environmental information processing, genetic information processing, human diseases, metabolism, and organismal systems (Figure 3B). Figure 3C lists the top 20 pathways, including phototransduction(map04744), NOD-like receptor signaling (map04621), inflammatory bowel disease (map05321), and progesterone-mediated oocyte maturation (map04914).

In the three groups of pituitary tissues, there were nine differentially expressed genes enriched in biological processes, cellular components, and molecular function. The differentially expresssed genes mainly enriched cellular processes (GO:0009987), metabolic processes (GO:0008152), biological regulation (GO:0065007), cellular component-like cell parts (GO:0044464), the cell (GO:0005623), and binding (GO:0005488) (Figure 3D). Next, we investigated the KEGG pathways in which all differentially expressed genes were significantly enriched in the hypothalamus. Thirty-seven enriched terms were involved in environmental information processing, human diseases, metabolism, and organismal systems (Figure 3E). Figure 3F lists the top 20 pathways, including inositol phosphate metabolism, TGF-β signaling, biosynthesis of unsaturated fatty acids, ovarian steroidogenesis, and GnRH signaling pathways.

**Figure 4:**
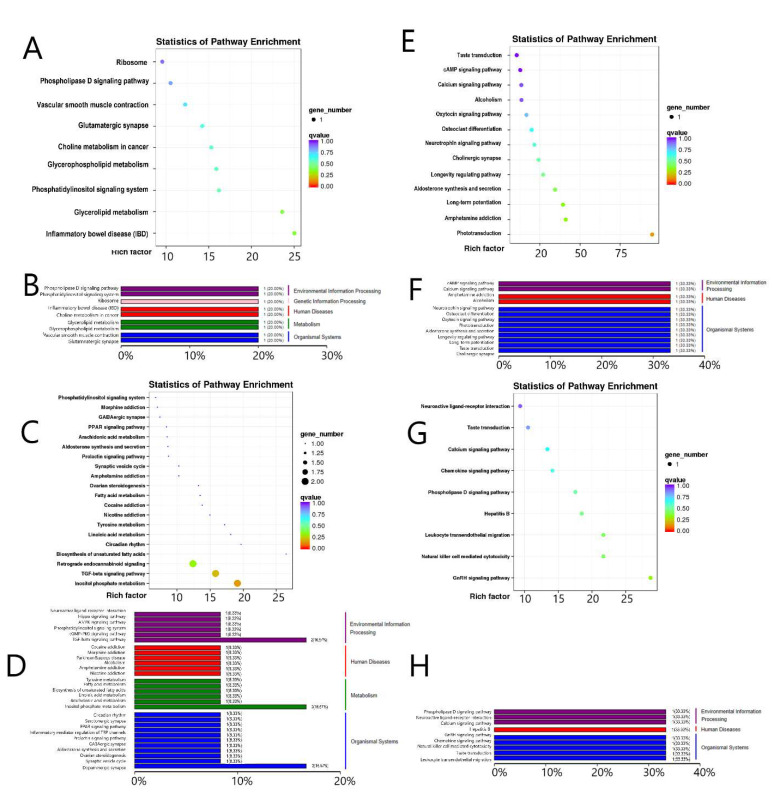
KEGG enrichment level of DEGs in the pituitary gland and hypothalamus at 18 and 30 months of age as compared with those at 6 months of age: (**A**,**B**) Pituitary gland at 18 months of age cas ompared with those at 6 months of age; (**C**,**D**) hypothalamus at 18 months of age as compared with those at 6 months of age; (**E**,**F**) pituitary gland at 30 months of age as compared with those at 6 months of age; (**G**,**H**) hypothalamus at 30 months of age as compared with those at 6 months of age.

In G3 vs. G1, DEGs were mainly enriched in gene information editing, metabolism, environmental information processing, and immune pathways (Figure 4A,B). In the hypothalamus, G2 vs. G4 involves differential gene functions mainly involved in metabolism, neuroimmunity, environmental information processing, biological systems (i.e., circadian rhythm, PPAR signaling, phosphatidylinositol signaling system, ovarian steroid production, aldosterone synthesis and secretion, GABA synapses, and prolactin signaling) (Figure 4C,D). In G5 vs. G1, differential gene functions were focused on biological system functions (i.e., phototransduction, aldosterone synthesis and secretion, longevity regulation, oxytocin signaling, and taste transduction), environmental information processing (i.e., the calcium signaling and cAMP signaling pathways), and immune disease pathways (Figure 4E,F). In G6 vs. G2, the hypothalamic differential gene functions were concentrated on biological system functions (GnRH signaling pathway and taste transduction), immune diseases, and environmental information processing (the calcium signaling and phospholipase D signaling pathways) (Figure 4G,H).

### 3.7. The Major Genes of the Pituitary Gland and Hypothalamus

According to the KEGG pathway enrichment results, five genes related to reproduction were screened out and listed in Table 2. In pituitary tissues, as compared with G1, CAMK4 was significantly downregulated in G5 which enriched the oxytocin signaling pathway. As compared with G3, HSP90AB1 was significantly upregulated in G5 which enriched progesterone-mediated oocyte maturation and the estrogen signaling pathway. In hypothalamus tissues, as compared with G2, TH, HSPB7, and LOC511936 were significantly upregulated in G4, TH was involved in prolactin pathway, HSPB7 and LOC511936 were engaged in steroid hormones synthesis pathway. As compared with G2, PTK2B was significantly downregulated in G6 which enriched the GnRH signaling pathway.

Other differentially expressed genes are listed in Table 3 and Table 4. In pituitary tissue, as compared with G1, TBX2 and SHANK2 were significantly downregulated in G3, PPP1R14A and HAPLN2 were significantly upregulated in G3. As compared with G1, TEX15 and CAMK4 were significantly downregulated in G5, ANGPTL7 and ORM1 were significantly upregulated in G5. As compared with G3, HSP90AB1 was significantly upregulated in G5 (Table 3).

In the hypothalamus, as listed in Table 4, as compared with G2, Rorb, SYNJ2, and TBR1 were downregulated in G4, and PCP4, KCNK3, and KCNK1 were significantly upregulated in G4. As compared with G2, only AGO3 was downregulated in G6, and CHRNA2, ORM1, and RLF were significantly upregulated in G6. As compared wit G4, CENPA and ZNF282 were downregulated in G6.

### 3.8. The Results of RT-qPCR

**Figure 5:**
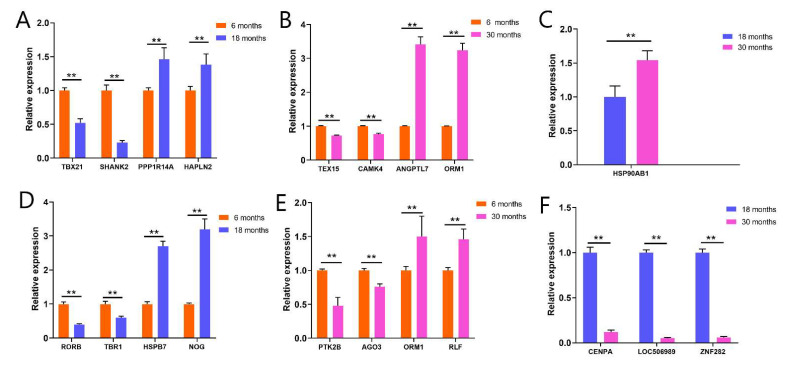
Differential gene expression levels in the pituitary and hypothalamus: (**A**) The results of G3 vs. G1; (**B**) the results of G5 vs. G1; (**C**) the result of G5 vs. G3; (**D**) the results of G4 vs. G2; (**E**) the results of G6 vs. G4; (**F**) the results of G6 vs. G4. (G1, G3, and G5 = 6-month-old, 18-month-old, and 30-month-old Angus cattle pituitary, respectively; G2, G4, and G6 = 6-month-old, 18-month-old, and 30-month-old Angus cattle hypothalamus, respectively). ** *p* < 0.01.

As shown in Figure 5, real-time quantitative PCR was used to verify the previous transcriptome sequencing results, and a total of 20 genes were detected. In pituitary tissues, as compared with G1, TBX21 and SHANK2 were significantly downregulated in G3, PPP1R14A and HAPLN2 were significantly upregulated in G3, TEX5 and CAMK4 were significantly downregulated in G5, and ANGPTL7 and ORM1 were significantly upregulated in G5. As compared with G3, HSP90AB1 was significantly upregulated in G5. In the hypothalamus, as compared with G2, RORB and TBR1 were significantly downregulated in G4, HSPB7 and NOG were significantly upregulated in G4, PTK2B and AGO3 were significantly downregulated in G6, and ORM1 and RLF were significantly upregulated in G4. As compared with G4, CENPA, LOC506989, and ZNF282 were significantly downregulated in G6. We compared RT-qPCR with the NGS results. As shown in Supplementary Figure S5, these genes showed the same change trend in RT-qPCR and NGS.

### 3.9. Protein Interaction Network Analysis

Fifty-six and 61 nodes and 1540 and 974 edges were included in the protein–protein interaction network of the pituitary gland and hypothalamus based on significant DEGs (minimum required interaction score >0.7). Analyzing the degree of centrality of the protein–protein interaction network showed that all genes were hub genes (degree = 55), and 40 hub genes (degree = 43) were obtained. The PRL family occupied a central position in the pituitary tissue, and the gene function of this family was mainly related to lysosomes. In the hypothalamus, the MRP family was in the core regulatory position, participating in the activation of the TGF-β signaling pathway and lysosome formation (Supplementary Figure S6).

## 4. Discussion

Previous studies have shown that at least 22,000 genes are present in the cattle genome, and 14,345 core group orthologs are shared among seven mammal species, but genes associated with lactation and immunity vary widely among species [24]. In our study, without calculating newly annotated genes, we detected 13,280 and 13,318 genes in the pituitary and hypothalamus tissues, therefore, genes expressed in the pituitary and hypothalamus accounted for 57.9% (13,280/22,000) and 58.1% (14,345/22,000) of the total number of genes in cattle.

The comparison of 6-month-old and 18-month-old Angus cattle showed that pituitary and hypothalamic tissue metabolism was active. This may be because calf growth and development in this period inevitably require a large energy supply. In the hypothalamus, TH enriched in the prolactin pathway. In this pathway, changes of TH directly affect tyrosine synthesis. TH is highly co-expressed with KiSS-1 neurons in the anterior ventricle-periventricular zone (AVPV/PeN). The AVPV/PeN TH neurons have dopaminergic activity. Dopamine signal transduction due to AVPV/PeN kisspeptin neurons may directly regulate GnRH secretion, thereby, regulating the neuroendocrine reproductive axis. The evidence suggests that some AVPV TH neurons project to PVN and activate prolactin neurons, thereby, increasing prolactin secretion [25].

In G6 vs. G2, PTK2b was significantly downregulated by Ca^2+^ in the calcium signaling pathway. In zebrafish models, PTK2b plays an important role in fertilization of zebrafish mother cells, and PTK2 is activated by fertilization-induced calcium [26]. PTK2 is also important for fertilization in mammals [27]. PTK2B is a downstream factor in this pathway, and its changes cause changes in other signal transductions, such as in the longevity regulation, long-term growth, and GnRH signaling pathways. PTK2b belongs to the protein tyrosine kinase family and plays an important role in various cellular processes.

In the pituitary, the role of CAMK4 in the calcium signaling pathway determines that changes in this pathway will directly affect fertilization, proliferation, learning, and memory. In the present study, CAMK4 was downregulated in G5 vs. G1 which enriched the oxytocin pathway. CAMK4 is an upstream regulator of nitric oxide synthase (NOS). Some studies have revealed the effect of CAMK4 on regulating NOS in neurological diseases [28], but the mechanism of its reproductive regulation remains unclear. CAMK4 downregulation directly caused PGC-1α downregulation, resulting in oxidative stress damage. Previous studies have shown that the oxidative state at the beginning of female reproduction affects the start and quality of the reproductive behavior [29]. Oxidative stress levels in previous reproductive behaviors may affect the quality of the next reproductive behavior. Activating CAMK4 via the calcium signaling pathway can downregulate PGC-1α and regulate oxidative stress in the body.

In G5 vs. G3, HSP90AB1 was upregulated which enriched the estrogen signaling and progesterone-mediated maturation pathway. In pigs, heat stress and lipopolysaccharides specifically regulate the protein abundance of ovarian HSP in the follicular and luteal phases. In cattle production, low estrus rates caused by heat stress may be related to HSP90AB1 [30]. Three differentially expressed genes, i.e., CENPA, LOC506989, and ZNF282, were found in G4 vs. G6. Although these three genes were not enriched in the KEGG pathway, early research has shown that CENPA is highly stable and is not supplemented after birth.

In this study, compared with the 6-month-old calves, the 18-month-old adults showed more enhanced sensory, metabolic, and physiological functions. This period is the main period of bodily development, but the growth rate of these functions gradually stabilizes with age until the body is fully developed. Between 18 months and 30 months of age, Angus cows showed better ovulation, which is a characteristic of sexual maturity. In 6-month-old pituitary tissue, only LOC101904796 was persistently highly expressed in 18-month-old and 30-month-old cows as compared with the 6-month-old cows, and this high expression did not differ between 18 and 30 months of age. LOC101904796 is a heterogeneous ribonucleoprotein responsible for the RNA recognition motif. No related reports have been published on this gene. According to the COG classification, we speculate that this gene is related to transcription and ribosome structure, which requires further research.

In production, factors such as photoperiod, dietary structure, and living environment affect carcass metabolism. Neurons in the brain that control metabolism are connected to reproductive neurons. The metabolic state affects the reproductive state. In our study, different differentially expressed genes were activated in multiple pathways such as metabolism, feeding, neurobehavior, and taste. ORM1 was co-expressed in both tissues, and the difference appeared in 30-month-old cattle as compared with 6-month-old cattle. The endoplasmic reticulum membrane protein family plays a central role in lipid homeostasis and protein quality control. In our study, ORM1 was significantly upregulated in 30-month-old cattle, likely related to the accelerated decomposition of ketone bodies in cows at this time. ORM1 upregulation may regulate lipid deposition and meat properties in cows.

The photoperiod must be controlled to improve bovine reproductive performance. This study showed activation of the circadian rhythm pathway in the hypothalamus in 18-month-old cattle. RORB is highly expressed in various animal models in the areas of the sensory organs, spinal cord, and brain that process circadian rhythm information [31]. In the pituitary glands of the 30-month-old cows, GNAT2 upregulation activated the photoperiod pathway. Although different genes regulate the photoperiod, the photoperiod affects the reproductive state.

Testosterone may play a role in ovulation in cows. Testosterone deficiency can effectively inhibit the surge of progesterone and LH, thereby, preventing ovulation [32]. In our study, TEX15 was significantly downregulated in the 30-month-old pituitary tissues. TEX11, TEX12, TEX14, and TEX15, are germ cell-specific genes expressed in the testes. TEX15 plays an important role in the correct assembly of synaptic complexes and meiosis. Interestingly, however, TEX15 expression was found in both the testes and ovaries [33]. In mouse models, loss of TEX15 gene function leads to early arrest of meiosis in male mice, which prevents meiotic recombination; however, this does not occur in female mice [34]. Downregulation of the TEX genes has been found in patients with azoospermia [35], confirming that this transcriptome is essential in the early stages of spermatogenesis. Although most functions of TEX15 are unknown, evidence suggests that TEX15, as a new susceptibility gene, is associated with breast cancer [36].

## 5. Conclusions

In general, we obtained mRNA expression profiles of the pituitary and hypothalamus of Angus cattle at different growth and developmental periods, providing transcriptome data for the study of the hypothalamic pituitary of Angus cattle. Through transcriptome data analysis, we found that in both hypothalamus and pituitary, the number of different expressed genes between 18 months and 6 months was greater than that between 30 months and 18 months. This suggests that gene expression in the hypothalamus and pituitary gland tend to stabilize in Angus cattle after adulthood. Consistent with our conjecture, both in the hypothalamus and pituitary, the differentially expressed genes between 18 months vs. 6 months were enriched in growth, development, and sexual maturity, while the differentially espressed genes between 30 months and 18 months were enriched in milk promotion. In the future, we need to conduct further functional verification of these differentially expressed genes to further understand the regulation mechanism of the hypothalamic pituitary gonad axis on Angus cattle reproduction.

## Figures and Tables

**Table 1:** Genes validated by RT-qPCR.

Gene	Full Name	Gene	Full Name
TBX21	T-box 21	TBR1	T-box, brain 1 factor
SHANK2	SH3 and multiple ankyrin repeat domains 2	HSPB7	Hsp20/α crystallin family
PPP1R14A	Protein phosphatase 1 regulatory inhibitor subunit 14A	NOG	Noggin
HAPLN2	Hyaluronan and proteoglycan link protein	PTK2B	Protein tyrosine kinase 2 β
TEX15	Testis expressed 15	AGO3	Argonaute RISC catalytic component 3
CAMK4	calcium/calmodulin dependent protein kinase IV	ORM1	Orosomucoid 1
ANGPTL7	Angiopoietin like 7	RLF	RLF zinc finger
ORM1	Orosomucoid 1	CENPA	Centromere protein A
HSP90AB1	Heat shock protein 90 alpha family class B member 1	LOC506989	Mitochondrial ribosomal protein L17-like
RORB	RAR-related orphan receptor B	ZNF282	Zinc finger protein 282

**Table 2:** Differentially expressed genes related to reproduction (G1, G3, and G5 = 6-month-old, 18-month-old, and 30-month-old Angus cattle pituitary; G2, G4, and G6 = 6-month-old, 18-month-old, and 30-month-old Angus cattle hypothalamus).

Group	Gene	FPKM	FPKM	FDR	log_2_FC	Regulated	Description
G5 vs. G1	CAMK4	14.772	40.334	0.000	−1.851	Down	Calcium/calmodulin dependent protein kinase IV
G5 vs. G3	HSP90AB1	27.882	0.170	0.000	7.228	Up	Hsp90 protein Heat shock protein 90 alphafamily class B member 1
G4 vs. G2	TH	7.633	27.451	0.005	−2.016	Down	Tyrosine hydroxylase
	LOC511936	11.263	5.386	0.022	2.100	Up	Cytochrome P450, family 2, subfamily J
G6 vs. G2	PTK2B	7.633	31.988	0.005	−2.016	Down	alpha

**Table 3:** Other major differentially expressed genes in pituitary gland (G1, G3, and G5 = 6-month-old, 18-month-old, and 30-month-old Angus cattle pituitary).

Group	Gene Name	G1	G3	G5	FDR	log_2_FC	Regulated	Description
G3 vs. G1	DGKH	2.859	0.679	1.872	0.004	−2.972	Down	Diacylglycerol kinase eta
	LOC101904138	0.666	0.032	0.339	0.007	−4.454	Down	Uncharacterized LOC101904138
	TPD52	2.334	33.140	8.349	0.009	3.775	Up	Tumor protein D52 like 3
	ZNF547	2.426	0.536	1.622	0.023	−3.205	Down	Zinc finger protein 547
	TBX21	1.996	0.083	2.905	0.015	−4.601	Down	T-box 21
	LOC101902860	38.591	1.351	25.463	0.010	−4.734	Down	Ribosomal L15
	SHISA3	4.420	0.273	8.643	0.032	−3.961	Down	Shisa family member 3
	HAPLN2	14.618	77.452	18.494	0.000	2.520	Up	Hyaluronan and proteoglycan link protein
	SHANK2	14.665	2.981	6.003	0.030	−2.428	Down	SH3 and multiple ankyrin repeat domains 2
	PPP1R14A	9.619	117.037	9.727	0.000	2.525	Up	Protein phosphatase 1 regulatory inhibitor subunit 14A
	LOC101904796	0.014	17.472	21.182	0.032	9.622	Up	Heterogeneous nuclear ribonucleoproteins A2/B1 pseudogene
	LOC534155	14.330	1.879	13.502	0.000	−2.859	Down	Immunoglobulin (CD79A) binding protein 1-like
G5 vs. G1	ORM1	0.013	22.558	35.752	0.000	10.936	Up	Orosomucoid 1
	LOC101906580	1.021	0.188	0.060	0.001	−4.021	Down	Uncharacterized LOC101906580
	GPATCH2L	5.110	7.325	9.942	0.000	1.600	Up	Phospholipase A2 group IVE
	CCDC168	0.000	0.166	0.383	0.000	Inf	Up	Coiled-coil domain containing 168
	ANGPTL7	0.628	2.566	4.627	0.000	2.788	Up	Angiopoietin like 7
	TEX15	2.016	0.541	0.484	0.012	−2.572	Down	Testis expressed 15
	GNAT2	0.000	0.648	0.689	0.000	Inf	Up	G protein subunit alpha transducin 2
	LYRM7	15.766	7.591	5.663	0.021	−1.716	Down	LYR motif containing 7
	PCDHGA7	0.215	0.910	2.116	0.000	3.262	Up	Protocadherin gammasubfamily A, 7
	LOC101904796	0.014	17.472	21.182	0.011	9.828	Up	Heterogeneous nuclear ribonucleoproteins A2/B1 pseudogene
	LOC100848679	3.066	2.119	0.967	0.000	−2.940	Down	DEAD-box ATP-dependent RNA helicase 30-like

**Table 4:** Other major differentially expressed genes in hypothalamus (G2, G4, and G6 = 6-month-old, 18-month-old, and 30-month-old Angus cattle hypothalamus).

Group	Gene Name	G2	G4	G6	FDR	log_2_FC	Regulated	Description
G4 vs. G2	RORB	2.771	0.887	1.385	0.041	−2.354	Down	RAR related orphan receptor B
	PCP4	199.707	645.127	419.042	0.010	1.654	Up	Purkinje cell protein 4
	LOC101904985	1.690	0.000	0.188	0.002	--	Down	Regulating synaptic membrane exocytosis protein 1-like
	SYNJ2	10.812	3.234	6.590	0.005	−1.855	Down	synaptojanin 2
	KCNK3	2.834	8.648	6.530	0.024	1.784	Up	Potassium two pore domain channel subfamily K member 3
	KCNF1	27.825	105.381	69.183	0.001	1.907	Up	Potassium two pore domain channel subfamily K member 1
	TBR1	13.041	1.779	5.804	0.044	−3.671	Down	T-box, brain 1 factor
	NKAIN3	8.259	2.222	5.496	0.000	−1.971	Down	sodium/potassium transporting ATPase interacting 3
	NPPB	2.539	13.985	7.234	0.044	2.455	Up	Natriuretic peptide B
	PLCH2	12.934	39.087	30.698	0.022	1.553	Up	Phospholipase C eta 2
	GABRD	20.752	56.832	46.509	0.040	1.538	Up	Gamma-aminobutyric acid type A receptor subunit delta
	DMKN	0.110	1.857	1.536	0.009	4.041	Up	Dermokine
	NOG	12.227	37.659	28.866	0.024	1.604	Up	Noggin
	KCNAB3	6.489	25.054	17.821	0.002	1.904	Up	KN motif and ankyrin repeat domains 3
	PCP4L1	67.687	310.150	217.532	0.047	2.215	Up	Purkinje cell protein 4 like 1
	ANKRD29	9.223	32.066	26.675	0.022	1.788	Up	Ankyrin repeat domain 29
	LOC101906058	50.638	13.883	37.250	0.011	−1.890	Down	Acyl-CoA desaturase-like
	NHSL2	4.488	1.153	3.759	0.007	−1.961	Down	NHS like 2
	SLC9A7	3.038	14.306	9.154	0.000	2.145	Up	Solute carrier family 9 member A7
	BMP8B	0.776	3.641	2.071	0.015	2.205	Up	Bone morphogenetic protein 8b
	CSMD2	4.889	1.299	3.543	0.025	−1.630	Down	CUB and Sushi multiple domains 2
	SHISA8	5.045	34.331	20.559	0.000	2.903	Up	Shisa family member 8
	CPLX1	39.927	121.167	89.915	0.018	1.569	Up	Complexin 1
	HSPB7	1.072	5.446	--	0.005	1.734	Up	Hsp20/alpha crystallin family
G6 vs. G2	CHRNA2	1.429	5.469	10.428	0.000	3.120	Up	Cholinergic receptor nicotinic alpha 2 subunit
	ORM1	0.039	15.589	56.033	0.003	10.333	Up	Orosomucoid 1
	VSIG10	2.858	13.395	25.630	0.000	3.010	Up	V-set and immunoglobulin domain containing 10
	SOX4	0.226	2.999	3.162	0.041	3.872	Up	SRY-box transcription factor 4
	RLF	3.370	4.590	6.354	0.027	1.858	Up	RLF zinc finger
	AGO3	6.829	4.594	1.811	0.000	−2.984	Down	argonaute RISC catalytic component 3
G6 vs. G4	CENPA	0.774	1.052	0.035	0.011	−4.415	Down	Centromere protein A
	ZNF282	2.947	5.137	1.750	0.034	−2.030	Down	Zinc finger protein 282

## Data Availability

All data involved in this article are original and available from the corresponding authors on reasonable request.

## Supplementary Materials

The following supporting information can be downloaded at: https://www.mdpi.com/article/10.3390/genes13060965/s1, Figure S1: Statistics for the variable splicing events; Figure S2: Distribution of reads in different regions of the genome; Figure S3: SNP density map and annotation classification diagram; Figure S4: Sample gene expression and overall distribution; Figure S5: The results of RT-qPCR were consistent with the NGS; Figure S6: Pituitary (left) and hypothalamus (right) DEGs network; Table S1: Grouping and numbering information; Table S2: Sequencing data and comparison results for each sample; Table S3: Statistics of the variable shear event structure; Table S4: Primers for real-time quantitative PCR; Table S5: SNP site statistics; Table S6: Numbers of differential genes; Table S7: New gene function annotation results; Table S8: Reproductive-related GO classification table involving new transcripts between groups; Table S9: Comparison of pituitary hormone genes and related receptor expression levels; Table S10: Specific functional transcription factors in the pituitary gland and hypothalamus.
