# Supplementary material for: Identification and Regulatory Network Analysis of Genes Related to Reproductive Performance in the Hypothalamus and Pituitary of Angus Cattle
Source: Genes (Basel). 2022 May 27;13(6):965. doi: 10.3390/genes13060965 (PMC9222274; doi:10.3390/genes13060965)
Supplement: Supplementary file 1 [file genes-13-00965-s001.zip › genes-1674439-supplementary.pdf]

# SUPPLEMENTARY

Supplementary Table S1. Grouping and numbering information

| Month | Pituitary | Hypothalamus |
|---|---|---|
| 6 | T01 T03 T05 (Group1) | T02 T04 T06 (Group2) |
| 18 | T07 T09 T11 (Group3) | T08 T10 T12 (Group4) |
| 30 | T13 T15 T17 (Group5) | T14 T16 T18 (Group6) |

Supplementary Table S2. Sequencing data and comparison results for each sample.

| Sample-ID | Total Reads | Mapped Reads | Uniq Mapped Reads | Multiple Map Reads | Reads Map to '+' | Reads Map to '-' | GC Content (%) | %≥Q30 |
|---|---|---|---|---|---|---|---|---|
| T1 | 57,118,252 | 54,816,325 (95.97%) | 54,042,135 (94.61%) | 774,190 (1.36%) | 27,353,429 (47.89%) | 27,385,142 (47.94%) | 48.21 | 94.70 |
| T2 | 50,699,016 | 48,523,637 (95.71%) | 47,830,518 (94.34%) | 693,119 (1.37%) | 24,215,110 (47.76%) | 24,227,642 (47.79%) | 48.48 | 94.39 |
| T3 | 44,959,694 | 43,008,790 (95.66%) | 42,405,126 (94.32%) | 603,664 (1.34%) | 21,455,108 (47.72%) | 21,489,408 (47.80%) | 48.02 | 94.27 |
| T4 | 46,484,518 | 44,533,701 (95.80%) | 43,884,485 (94.41%) | 649,216 (1.40%) | 22,209,686 (47.78%) | 22,249,549 (47.86%) | 48.19 | 94.25 |
| T5 | 47,866,648 | 45,900,604 (95.89%) | 45,231,684 (94.50%) | 668,920 (1.40%) | 22,896,323 (47.83%) | 22,925,322 (47.89%) | 48.10 | 94.19 |
| T6 | 42,429,662 | 40,730,396 (96.00%) | 40,122,977 (94.56%) | 607,419 (1.43%) | 20,321,568 (47.89%) | 20,344,945 (47.95%) | 48.57 | 94.79 |
| T7 | 42,093,036 | 40,343,568 (95.84%) | 39,783,217 (94.51%) | 560,351 (1.33%) | 20,117,091 (47.79%) | 20,152,776 (47.88%) | 48.31 | 94.55 |
| T8 | 53,370,874 | 51,268,347 (96.06%) | 50,525,761 (94.67%) | 742,586 (1.39%) | 25,568,874 (47.91%) | 25,615,228 (47.99%) | 48.07 | 94.64 |
| T9 | 45,486,310 | 43,599,130 (95.85%) | 42,928,790 (94.38%) | 670,340 (1.47%) | 21,756,027 (47.83%) | 21,769,779 (47.86%) | 48.37 | 94.61 |
| T10 | 48,425,134 | 46,417,437 (95.85%) | 45,678,600 (94.33%) | 738,837 (1.53%) | 23,138,023 (47.78%) | 23,167,391 (47.84%) | 48.70 | 94.64 |
| T11 | 45,893,680 | 43,625,973 (95.06%) | 42,757,833 (93.17%) | 868,140 (1.89%) | 21,709,308 (47.30%) | 21,752,593 (47.40%) | 49.17 | 94.26 |
| T12 | 45,320,976 | 43,574,262 (96.15%) | 42,972,962 (94.82%) | 601,300 (1.33%) | 21,740,072 (47.97%) | 21,769,970 (48.04%) | 48.41 | 94.77 |
| T13 | 58,017,574 | 55,713,072 (96.03%) | 54,950,762 (94.71%) | 762,310 (1.31%) | 27,807,615 (47.93%) | 27,826,194 (47.96%) | 48.20 | 94.48 |
| T14 | 48,262,590 | 46,502,866 (96.35%) | 45,896,580 (95.10%) | 606,286 (1.26%) | 23,220,420 (48.11%) | 23,220,923 (48.11%) | 48.00 | 94.48 |
| T15 | 46,909,660 | 45,042,180 (96.02%) | 44,412,368 (94.68%) | 629,812 (1.34%) | 22,471,758 (47.90%) | 22,503,901 (47.97%) | 48.15 | 94.31 |
| T16 | 49,442,698 | 47,442,833 (95.96%) | 46,694,886 (94.44%) | 747,947 (1.51%) | 23,662,959 (47.86%) | 23,681,663 (47.90%) | 48.34 | 94.68 |

| | | | | | | | | |
|---|---|---|---|---|---|---|---|---|
| T17 | 46,414,634 | 44,486,879 (95.85%) | 43,857,110 (94.49%) | 629,769 (1.36%) | 22,190,044 (47.81%) | 22,220,732 (47.87%) | 47.61 | 94.56 |
| T18 | 44,376,678 | 42,383,143 (95.51%) | 41,791,234 (94.17%) | 591,909 (1.33%) | 21,136,472 (47.63%) | 21,174,333 (47.72%) | 47.89 | 94.78 |

Total reads: number of clean reads, calculated as single-ended.
Mapped reads: number of reads compared with the reference genome and their percentage in the clean reads.
Unique mapped reads: number of reads compared with the unique position of the reference genome and percentage in the clean reads.
Multiple map reads: Comparison of the number of reads at multiple locations in the reference genome and their percentage in clean reads.
Reads map to “+”: number of reads compared with the positive strand of the reference genome and their percentage in clean reads. Reads map to “-“: number of reads compared with the negative strand of the reference genome and their percentage in clean reads.
GC content: content of the clean data; GC is the percentage of G and C bases in the clean data to the total bases.
≥Q30%: percentage of bases with a clean data quality value ≥30.

Supplementary Table S3. Statistics of the variable shear event structure.

| Event ID | Event Type | Gene ID | Chrom | Event Start | Event End | Event Pattern | Strand |
|---|---|---|---|---|---|---|---|
| 1000001 | TSS | gene100 | AC_000158.1 | 9607340 | 9607574 | 9607574 | + |
| 1000002 | TTS | gene100 | AC_000158.1 | 9920035 | 9921259 | 9920035 | + |
| 1000003 | SKIP_ON | gene100 | AC_000158.1 | 9798277 | 9798333 | 9795702,9798277-<br>9798333,9811726 | + |
| 1000004 | SKIP_OFF | gene100 | AC_000158.1 | 9798277 | 9798333 | 97,957,029,811,726 | + |
| 1000005 | MSKIP_ON | gene100 | AC_000158.1 | 9795535 | 9798333 | 9777740,9795535-<br>9795702,9798277-<br>9798333,9811726 | + |
| 1000006 | MSKIP_OFF | gene100 | AC_000158.1 | 9795535 | 9798333 | 97,777,409,811,726 | + |
| 1000007 | SKIP_ON | gene100 | AC_000158.1 | 9795535 | 9795702 | 9777740,9795535-<br>9795702,9811726 | + |
| 1000008 | SKIP_OFF | gene100 | AC_000158.1 | 9795535 | 9795702 | 97,777,409,811,726 | + |
| 1000009 | TSS | gene1006 | AC_000158.1 | 133395622 | 133395742 | 133395622 | - |

Event ID: AS event number.

Event Type: AS event type.

Gene ID: gene number.

Chrom: chromosome number.

Event Star: AS event start position.

Event End: AS event end position.

Event pattern: AS event characteristics.

Strand: gene positive and negative chain information.

Supplementary Table S4. Primers for real-time quantitative PCR.

| Target Name | | Primer |
|---|---|---|
| PPP1R11 | F | CAGAAAAGACAGAAGGGTGCC |
| | R | CGTTTCCGAAGTTTGATGGTTA |
| TBX21 | F | GCCTACCAGAATGCCGAGA |
| | R | TCAACGGAAGGATACATGGACT |
| SHANK2 | F | ACATCTGGACCCTCTACAACC |
| | R | CTTCAGTTCCTTATCTCCACCT |
| PPP1R14A | F | ACATGCCCGATGAGGTCAAC |
| | R | AGTCACTGGATTTTACGGCTT |
| HAPLN2 | F | CCACCTACGCCCAGCAGTACC |
| | R | AGCCCGCGTTACACCAGTCCA |
| TEX15 | F | CTCTTCAAGTATACGCGT |
| | R | TGTCTTTACTTTCTTCGGTT |
| CAMK4 | F | TCTCTGGTCGCTTCTATCGCT |
| | R | CCTCCTCGTCACTCCT |
| ANGPTL7 | F | ACGGAATAGCTATCGCCT |
| | R | GCAGCAGTTGTACCAGTAT |
| ORM1 | F | GTCGGCTTTCTTTTACCTT |
| | R | CAAAGTGTTCTCTGTCC |
| HSP90AB1 | F | CACTCCAACCGCATCTACC |
| | R | CTCCTCTACCGTCACCTC |
| RORB | F | CCTGACATCCGTACCCAA |
| | R | ATTTCGGTCATTGTTATCCCT |
| TBR1 | F | GCCCTCCTCTATCAAGTCCAT |
| | R | TTTGGCCTGCTCGTAAAT |
| HSPB7 | F | CTTCATGCGTCCCCACTCGG |
| | R | CCACAGCAAACTCATAGGCGTCTC |
| NOG | F | ACCTCATCGAACACACGGACC |
| | R | AGCAGCGTCTCGTTCAGATCC |
| ORM1 | F | GTCGGCTTTCTTTTACCTT |
| | R | CAAAGTGTTCTCTGTCC |
| PTK2B | F | CCTGAAAGAAGACAAGACCAC |
| | R | CCATCCCCTCGCTGTCC |
| AGO3 | F | GGCATCTCAAGAACACCT |
| | R | TATCTCCGACACGATTGACC |
| RLF | F | GAGGCATTACAAACGCAC |
| | R | GCTCCTCCTTAATTTTGGT |
| CENPA | F | GCCCCTTCTGCCGACTG |
| | R | AAATGCTTCTGGCGCCTC |
| LOC506989 | F | TCAACTGCTGGCCCCTCG |
| | R | TGGTGTCTCTTCGTGCTA |
| ZNF282 | F | ACATCCCAACGTACCCC |
| | R | CTGGTCCTCCTGCTTTATC |

Supplementary Table S5. SNP site statistics.

| Sample-ID | SNP Number | Genic SNP | Intergenic SNP | Transition | Transversion | Heterozygosity |
|---|---|---|---|---|---|---|
| T1 | 247,191 | 203,899 | 43,292 | 72.33% | 27.67% | 40.68% |
| T2 | 203,152 | 169,538 | 33,614 | 71.97% | 28.03% | 40.50% |
| T3 | 228,495 | 189,837 | 38,658 | 71.96% | 28.04% | 45.37% |
| T4 | 239,243 | 198,611 | 40,632 | 72.03% | 27.97% | 45.40% |
| T5 | 198,388 | 163,998 | 34,390 | 72.10% | 27.90% | 38.80% |
| T6 | 140,088 | 113,535 | 26,553 | 72.12% | 27.88% | 37.68% |
| T7 | 186,525 | 154,055 | 32,470 | 72.28% | 27.72% | 42.71% |
| T8 | 212,891 | 174,209 | 38,682 | 72.22% | 27.78% | 43.93% |
| T9 | 173,482 | 144,102 | 29,380 | 72.03% | 27.97% | 38.03% |
| T10 | 130,330 | 105,090 | 25,240 | 72.08% | 27.92% | 39.99% |
| T11 | 75,571 | 57,997 | 17,574 | 72.10% | 27.90% | 40.49% |
| T12 | 196,402 | 161,703 | 34,699 | 72.14% | 27.86% | 41.42% |
| T13 | 223,090 | 185,434 | 37,656 | 72.05% | 27.95% | 41.72% |
| T14 | 199,483 | 165,311 | 34,172 | 72.19% | 27.81% | 40.20% |
| T15 | 194,608 | 162,245 | 32,363 | 72.07% | 27.93% | 39.63% |
| T16 | 166,754 | 136,553 | 30,201 | 72.08% | 27.92% | 39.16% |
| T17 | 196,132 | 161,964 | 34,168 | 71.92% | 28.08% | 40.92% |
| T18 | 201,488 | 165,848 | 35,640 | 72.03% | 27.97% | 41.01% |

SNP Number: total number of SNP sites.

Genic SNP: total number of SNP loci in the gene region.

Intergenic SNP: total number of SNP loci in the intergenic region.

Transition: percentage of conversion-type SNP sites in the total number of SNP sites. Transversion: percentage of the number of transversion-type SNP sites in the total number of SNP sites.

Heterozygosity: percentage of the number of heterozygous SNP sites in the total number of SNP sites.

Supplementary Table S6. Numbers of differential genes.

| Parameters | Group | DEGs total | DEGs up | DEGs down |
|---|---|---|---|---|
| DESeq EBSeq FDR<0.05 FC⩾2 | G3vsG1 | 18 | 4 | 14 |
| | G5vsG1 | 18 | 11 | 7 |
| | G5vsG3 | 3 | 3 | 0 |
| | G4vsG2 | 36 | 24 | 12 |
| | G6vsG2 | 12 | 7 | 5 |
| | G6vsG4 | 3 | 0 | 3 |
| | G1vsG2 | 402 | 258 | 144 |
| | G3vsG4 | 6 | 0 | 6 |
| | G5vsG6 | 5 | 2 | 3 |

Parameters: testing software used DESeq and EBSeq; the evaluation standards were FDR<0.05 and FC⩾2.

Supplementary Table S7. New gene function annotation results

| Annotated databases | New Gene Number |
|---|---|
| COG | 179 |
| GO | 2,933 |
| KEGG | 2,012 |
| KOG | 896 |
| Pfam | 863 |
| Swiss-Prot | 1,032 |
| eggNOG | 2,585 |
| Nr | 4,053 |
| All | 4,072 |

Supplementary Table S8. Reproductive-related GO classification table involving new transcripts between groups

| Group | GO annotation (Biological process) | GO Classification Number | New transcription | Differential Transcriptions |
|---|---|---|---|---|
| G3vsG1 | reproduction | GO:0000003 | 56 | 7 |
| | reproductive process | GO:0022414 | 56 | 7 |
| G5vsG1 | reproduction | GO:0000003 | 56 | 9 |
| | reproductive process | GO:0022414 | 56 | 9 |
| G5vsG3 | reproduction | GO:0000003 | 56 | 8 |
| | reproductive process | GO:0022414 | 56 | 8 |
| G4vsG2 | reproduction | GO:0000003 | 56 | 6 |
| | reproductive process | GO:0022414 | 56 | 6 |
| G6vsG2 | reproduction | GO:0000003 | 56 | 6 |
| | reproductive process | GO:0022414 | 56 | 6 |
| G6vsG4 | reproduction | GO:0000003 | 56 | 6 |
| | reproductive process | GO:0022414 | 56 | 6 |

Supplementary Table S9. Comparison of pituitary hormone genes and related receptor expression levels

| Gene | Description | FPKM in Groups | | | | | | Ratio of groups in FPKM | | | |
|---|---|---|---|---|---|---|---|---|---|---|---|
| | | G1 | G2 | G3 | G4 | G5 | G6 | G3/G1 | G5/G1 | G4/G2 | G6/G2 |
| Pomc | Proopiomelanocortin | 3.635 | 4.983 | 18.480 | 6.265 | 4.875 | 4.385 | 5.084 | 1.341 | 1.257 | 0.880 |
| PRL | Prolactin | 0.007 | 0.035 | 0.018 | 0.087 | 0.076 | 0.068 | 2.405 | 10.255 | 2.485 | 1.957 |
| LHb | Luteinizing hormone beta polypeptide | 1.344 | 1.570 | 2.035 | 0.932 | 0.950 | 1.616 | 1.514 | 0.707 | 0.593 | 1.029 |
| TSHb | Thyroid-stimulating hormone, β-subunit | 0.029 | 0.056 | 0.066 | 0.026 | 0.033 | 0.123 | 2.261 | 1.155 | 0.459 | 2.180 |
| Ghdc | GH3 domain containing | 6.836 | 6.703 | 7.838 | 5.417 | 6.117 | 12.531 | 1.147 | 0.895 | 0.808 | 1.870 |
| GHR | Growth hormone receptor | 1.034 | 1.956 | 1.905 | 1.606 | 2.306 | 1.633 | 1.843 | 2.230 | 0.821 | 0.835 |
| GHSR | Growth hormone secretagogue receptor | 4.396 | 3.147 | 3.064 | 3.336 | 2.771 | 1.904 | 0.697 | 0.630 | 1.060 | 0.605 |
| PRLR | Prolactin receptor | 0.897 | 0.853 | 0.759 | 0.943 | 0.742 | 0.983 | 0.846 | 0.827 | 1.105 | 1.153 |

Supplementary Table S10. Specific functional transcription factors in the pituitary gland and hypothalamus

| Gene | Description | FPKM in Groups | | | | | | Ratio of groups in FPKM | | | |
|---|---|---|---|---|---|---|---|---|---|---|---|
| | | G1 | G2 | G3 | G4 | G5 | G6 | G3/G1 | G5/G1 | G4/G2 | G6/G2 |
| LHX2 | LIM homeobox 2 | 9.803 | 15.276 | 13.683 | 12.961 | 15.320 | 14.882 | 1.396 | 1.563 | 0.848 | 0.974 |
| LHX6 | LIM homeobox 6 | 0.155 | 4.952 | 0.195 | 1.106 | 0.151 | 1.579 | 1.258 | 0.972 | 0.223 | 0.319 |
| DLX2 | distal less homeobox 2 | 19.235 | 5.404 | 11.846 | 16.663 | 20.889 | 14.105 | 0.616 | 1.086 | 3.083 | 2.610 |
| TBX2 | T-box transcription factors | 0.851 | 1.217 | 1.390 | 1.452 | 1.861 | 1.282 | 1.634 | 2.187 | 1.193 | 1.053 |
| SOX9 | SRY-box 9 | 3.426 | 5.672 | 3.967 | 2.663 | 4.789 | 3.787 | 1.158 | 1.398 | 0.470 | 0.668 |
| SOX8 | SRY-box 8 | 13.662 | 26.216 | 31.092 | 15.829 | 16.550 | 19.277 | 2.276 | 1.211 | 0.604 | 0.735 |
| SOX2 | SRY-box 2 | 38.963 | 41.830 | 37.558 | 37.433 | 46.780 | 42.536 | 0.964 | 1.201 | 0.895 | 1.017 |
| GATA2 | GATA binding protein 2 | 0.836 | 1.555 | 1.178 | 1.060 | 1.453 | 1.233 | 1.408 | 1.738 | 0.681 | 0.793 |

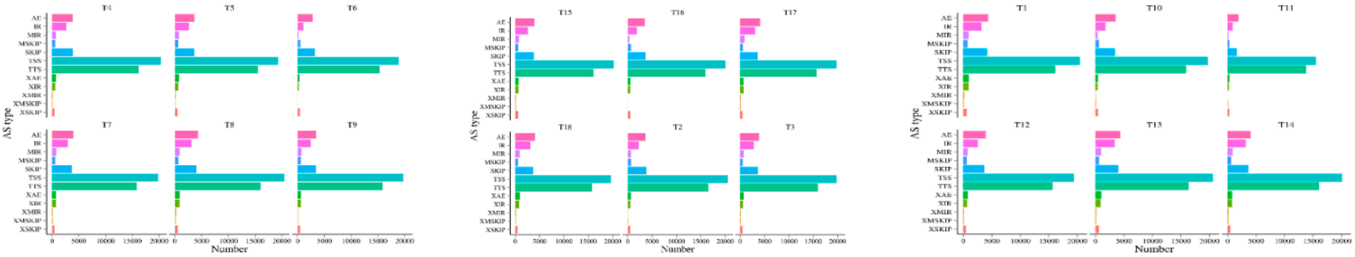


Supplementary Figure S1. Statistics for the variable splicing events.

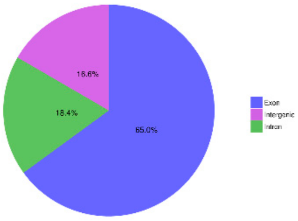


Supplementary Figure S2. Distribution of reads in different regions of the genome. Exons are blue; introns are green; intergenic regions are purple.

(a) (b) (c)

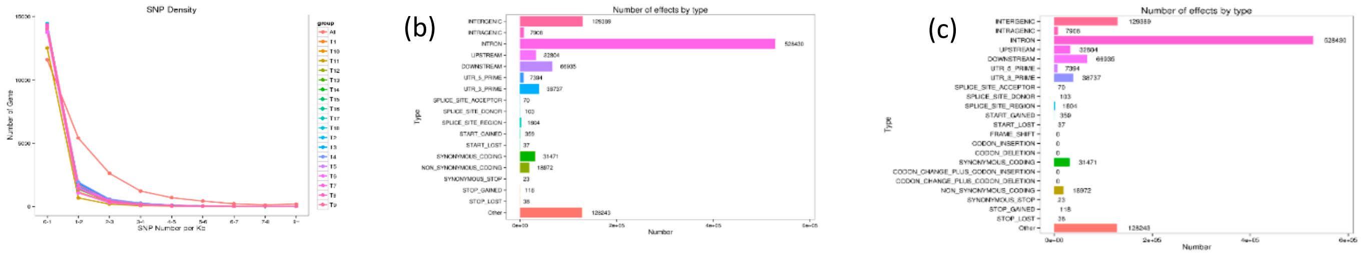


Supplementary Figure S3. SNP density map and annotation classification diagram. (a) SNP density map; annotation classification diagram. (b) SNP annotation classification diagram. (c) Indel annotation classification diagram. The vertical axis is the area or type where the SNP/InDel is located, and the horizontal axis is the number of categories.

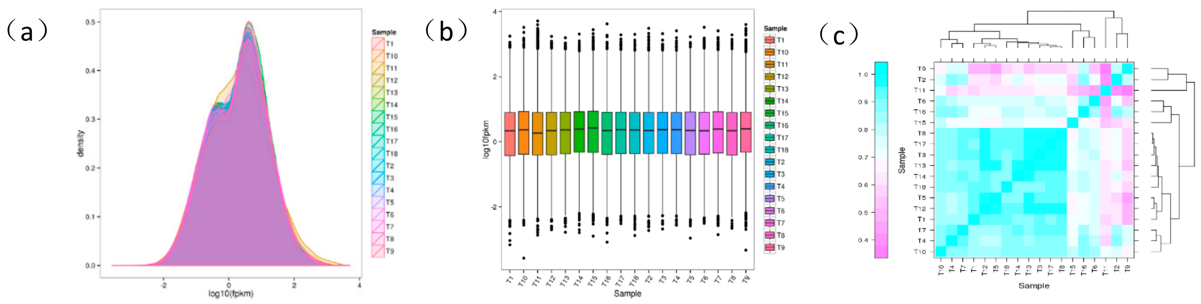


Supplementary Figure S4. Sample gene expression and overall distribution. (a) Comparison chart of FPKM density distribution in each sample. (b) FPKM box line diagram of each sample. The graph measures the expression level of each sample from the overall discrete perspective of the expression level. (c) Heatmaps with clustering of analyzed samples.

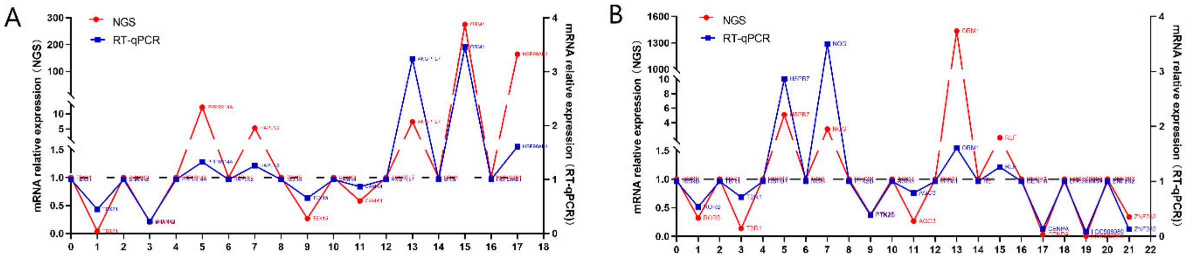


Supplementary Figure S5. The results of RT-qPCR were consistent with the NGS. A was the pituitary gland, B was the hypothalamus, red was the results of NGS, blue was the results of RT-QPCR.

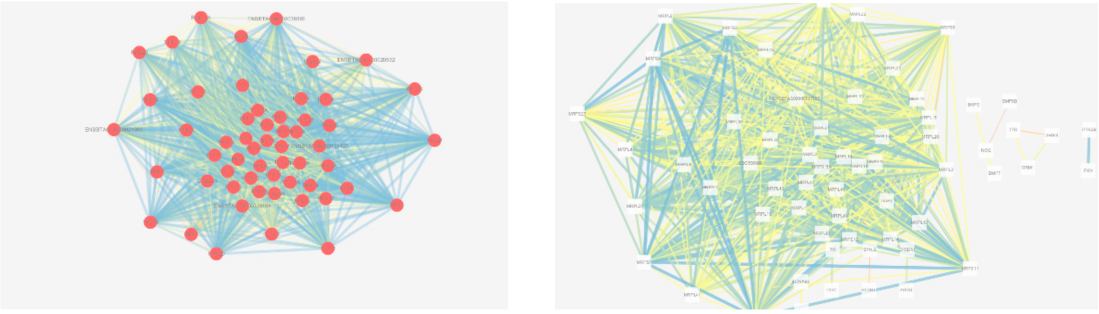

Supplementary Figure S6. Pituitary (left) and hypothalamus (right) DEGs network.
